# Genome Sequences of Five Brucella canis Strains Isolated from Different Countries throughout the World

**DOI:** 10.1128/MRA.01065-18

**Published:** 2018-10-11

**Authors:** Guillaume Girault, Acácia Ferreira Vicente, Yannick Corde, Mateus Souza Ribeiro Mioni, Lara Borges Keid, Maryne Jay, Jane Megid, Virginie Mick

**Affiliations:** a EU/OIE/FAO & National Reference Laboratory for Animal Brucellosis, Animal Health Laboratory, Paris-Est University/Anses, Maisons-Alfort, France; b Molecular Biology Laboratory, Department of Veterinary Hygiene and Public Health, FMVZ, UNESP, Botucatu, Brazil; c Department of Veterinary Medicine, University of Animal Science and Food Engineering, USP, Pirassununga, Brazil; Iowa State University

## Abstract

Canine brucellosis is a major underestimated zoonosis that remains endemic in many areas of the world. A recent phylogeographic investigation including 53 Brucella canis field isolates revealed the existence of two major lineages worldwide. Here, we report genome sequencing of 5 representative isolates of different clades identified in this study.

## Abstract (precis)

Canine brucellosis is a major underestimated zoonosis that remains endemic in many areas of the world. A recent phylogeographic investigation including 53 Brucella canis field isolates revealed the existence of two major lineages worldwide.

## ANNOUNCEMENT

Brucellosis, caused by species of the Brucella genus, is a major worldwide zoonosis affecting a large host range, from cattle to humans (1). *Brucella* is a genus in constant evolution with a large number of species (2–4). Brucella canis, responsible for canine brucellosis, is usually isolated from dogs, and it can occasionally cause infection in humans (1). Canine brucellosis is probably neglected in animals and humans. Recently, we reported a comparative phylogeographic investigation of 53 B. canis field strains, mainly isolated from Brazil, where the disease remains endemic (5). Worldwide strains were subdivided into two main lineages and into different subclades.

Here, we report genome sequences of 5 representative strains of the B. canis phylogeny, which were isolated from dogs; 2 strains were isolated from Europe and 3 from Brazil (Table 1). Genomic DNA was extracted using the High Pure PCR template prep kit (Roche Diagnostics, France) according to the manufacturer’s instructions. Libraries were prepared with the Nextera XT sample prep kit (Illumina, Inc.). Whole-genome sequencing was performed on a MiSeq (Illumina, Inc.) platform with 250-bp paired-end reads. Raw reads were checked for quality by FastQC v0.11.5 and trimmed using Trimmomatic 0.36 (phred, 33; minimum length, 50 bp). *De novo* assembly was performed using SPAdes 3.7.1 (iterative k-mer values of 55, 77, 99, and 127). The different assembly values are indicated in Table 1. Assemblies resulted in genome sizes ranging from 3,255,541 to 3,294,648 bp, with an average G+C content of 57.27% according to the Brucella genus. The average contig coverage was 41-fold. A consistent automatic annotation was generated by the Rapid Annotation using Subsystem Technology RASTtk at the PATRIC Bioinformatics resource center. An average of 3,306 coding DNA sequences (CDS) were predicted.

**TABLE 1:** *Brucella canis* genomes sequenced in this study

Strain	Country of origin	Year	Host	SRA accession no.^*a*^	ENA accession no.^*b*^	Genome size (bp)	No. of contigs >1,000 bp	Mean coverage (×)	Contig *N*_50_ (bp)	No. of CDS (RAST)
96-9626	Spain	1996	Dog	ERR2136545	UEXH01000000	3,294,648	26	47	316,480	3,307
07-2859-6070	Brazil	1998	Dog	ERR2136546	UEXG01000000	3,292,531	29	46	211,698	3,310
07-2859-6071	Brazil	1995	Dog	ERR2136547	UEXI01000000	3,290,866	25	39	299,689	3,317
09-369-776-1	Finland	2009	Dog	ERR2136548	UFQW00000000	3,255,541	33	38	208,450	3,282
10469	Brazil	2005	Dog	ERR2136549	UEXJ01000000	3,293,240	24	34	348,106	3,313

a Raw reads.

b Contigs.

The genome sequences of 5 B. canis isolates from South America and Europe reported here are a valuable source of information for studying epidemiology of this underestimated infection.

### Data availability.

This whole-genome sequencing project (PRJEB22763) has been deposited in the European Nucleotide Archive (ENA) under the accession numbers UFQW00000000 (UFQW01000001 to UFQW01000033) (strain 09-369-776-1), UEXJ01000000 (UEXJ01000001 to UEXJ01000024) (strain 10469), UEXH01000000 (UEXH01000001 to UEXH01000026) (strain 96-9626), UEXI01000000 (UEXI01000001 to UEXI01000025) (strain 07-2859-6071), and UEXG01000000 (UEXG01000001 to UEXG01000029) (strain 07-2859-6070). The versions described in this paper are the first versions.
